# Measuring mortality and the burden of adult disease associated with adverse childhood experiences in England: a national survey

**DOI:** 10.1093/pubmed/fdu065

**Published:** 2014-08-30

**Authors:** M.A. Bellis, K. Hughes, N. Leckenby, K.A. Hardcastle, C. Perkins, H. Lowey

**Affiliations:** 1 Centre for Public Health, World Health Organization Collaborating Centre for Violence Prevention, Liverpool John Moores University, 15-21 Webster Street, Liverpool L3 2ET, UK; 2 Public Health Wales, Hadyn Ellis Building, Maindy Road, Cardiff CF24 4HQ, UK; 3 Knowledge and Intelligence Team (North West), Public Health England, 15-21 Webster Street, Liverpool L3 2ET, UK; 4 Blackburn with Darwin Borough Council, Specialist Public Health Directorate, 10 Duke Street, Blackburn BB2 1DH, UK

**Keywords:** children, chronic disease, morbidity and mortality

## Abstract

**Background:**

ACE (adverse childhood experience) studies typically examine the links between childhood stressors and adult health harming behaviours. Using an enhanced ACE survey methodology, we examine impacts of ACEs on non-communicable diseases and incorporate a proxy measure of premature mortality in England.

**Methods:**

A nationally representative survey was undertaken (*n* = 3885, aged 18–69, April–July 2013). Socio-demographically controlled proportional hazards analyses examined the associations between the number of ACE categories (<18 years; e.g. child abuse and family dysfunction such as domestic violence) and cancer, diabetes, stroke, respiratory, liver/digestive and cardiovascular disease. Sibling (*n* = 6983) mortality was similarly analysed as a measure of premature mortality.

**Results:**

Of the total, 46.4% of respondents reported ≥1 and 8.3% ≥4 ACEs. Disease development was strongly associated with increased ACEs (e.g. hazard ratios, HR, 0 versus ≥4 ACEs; cancer, 2.38 (1.48–3.83); diabetes, 2.99 (1.90–4.72); stroke, 5.79 (2.43–13.80, all *P* < 0.001). Individuals with ≥4 ACEs (versus no ACEs) had a 2.76 times higher rate of developing any disease before age 70 years. Adjusted HR for mortality was strongly linked to ACEs (≥4 versus 0 ACEs; HR, 1.97 (1.39–2.79), *P* < 0.001).

**Conclusions:**

Radically different life-course trajectories are associated with exposure to increased ACEs. Interventions to prevent ACEs are available but rarely implemented at scale. Treating the resulting health costs across the life course is unsustainable.

## Introduction

Globally, health strategies are increasingly recognizing the link between early childhood experiences and health outcomes across the life course.^1,2^ The importance of early life experiences is so pronounced that in Europe, the World Health Organization's (WHO) review of social determinants and health states that ‘The highest priority is for countries to ensure a good start to life for every child’.^3^ Poor quality childhoods are often associated with societal level factors such as deprivation and inequities.^4–7^ However, there is increasing evidence that specific childhood experiences increase the risk of individuals adopting health-harming behaviours and developing chronic ill health in later life.^8–10^ A well-defined set of adverse childhood experiences (ACEs) linked to poor outcomes has been identified and refined as a tool for measuring childhood adversity and its impact on population health.^11–13^ These include exposure directly to child abuse (sexual, verbal and physical) and more broadly to family dysfunction including domestic violence, parental separation and household members with substance abuse, poor mental health or incarceration. Increasing exposure to such childhood adversity has been associated with escalating risk of problems including substance use, violence, sexual risk-taking and suicide ideation.^8,10,14,15^ Increasingly, the impact of ACEs on longer term morbidity from non-communicable disease (NCD) and on premature mortality is becoming apparent.^9,16–19^

In Europe, WHO's ‘European report on preventing child maltreatment^20^ highlights the importance of preventing ACEs along with the need for routine surveys to determine their prevalence and impacts on health, and enable the effects of prevention activity to be monitored. The *European Child Maltreatment Action Plan* is intended to establish these as priorities across the region. ACE surveys allow rapid data collection from adults on retrospective childhood experiences and adult health outcomes, and thus provide a valuable source of intelligence for policy-makers. Despite a proliferation of ACE surveys^20^ however, a key restriction is their omission of individuals who have died between experiencing ACEs and study data collection—years, sometimes decades, later. Critically, such mortality is in part a function of exposure to ACEs.^9,16^ Thus, around a decade after the initial San Diego ACE study, linkage of baseline data with mortality records estimated that individuals with ≥6 ACEs (versus none) were 1.7 times more likely to die ≤75 years.^17^ Of individuals who died of lung cancer, those with ≥6 ACEs died almost 13 years earlier than those with no ACEs.^18^ Linkage of data from a separate birth cohort and mortality records found increased risks of premature mortality among those living with ≥2 ACE-like issues (at ages 7, 11 or 16; data collected from parents and teachers) reached 57% for males and 80% for females.^19^

Few countries have large-scale, established cohort studies examining ACEs that can be linked to health and mortality records, and even where available these are often limited to specific population groups and generations. Consequently, despite their clear benefits, cohort studies do not typically permit measurement of how ACEs and their associations with health affect successive generations across the entire population. To allow some measure of premature mortality within standard retrospective ACE studies, siblings' mortality might be used as a proxy. Siblings often experience similar indirect childhood environmental factors including poverty, parental separation and household substance abuse, while siblings of abused children will often be exposed to violence themselves, either directly^21,22^ or as a witness. Here we undertake a national ACE survey but with inclusion of measures of sibling mortality. We examine how ACEs impact on risks of developing major long-term diseases in study participants. Additionally, we examine sibling mortality and use this to quantify ACE impacts over and above morbidity reported by participants. Finally, for those with the poorest health outcomes (males from the most deprived communities), we combine morbidity and mortality associated with increasing ACE counts to provide a more complete measure of the impact of early life adversity on long-term health.

## Methods

### Questionnaire

The questionnaire examined participant demographics, health-harming behaviours (e.g. smoking, alcohol misuse; not addressed here), chronic diseases and ACEs. Identification of ACEs used the short ACE tool developed by Kaiser Permanente and the Centers for Disease Control and Prevention.^23^ The tool includes 11 questions on childhood exposure to abuse and family dysfunction occurring <18 years of age. These form nine categories of ACE covering: physical, verbal and sexual abuse; parental separation; exposure to domestic violence and growing up in a household with mental illness, alcohol abuse, drug abuse or incarceration (see Supplementary data, Box). For major disease categories (respiratory disease; cancer; diabetes type 2; cardiovascular disease (CVD); stroke and liver/digestive disease), individuals reported if they had ever been diagnosed with each condition by a doctor or nurse, and if so when the first diagnosis had occurred. Prompts were provided on conditions included in individual categories (e.g. respiratory disease: chronic bronchitis, emphysema, chronic obstructive pulmonary disease; CVD: coronary heart disease, heart attack). Individuals also completed the Short Warwick-Edinburgh Mental Well-being Scale,^24^ although these variables are not reported in this paper. Respondents were also asked the number of siblings they lived with during childhood. Individuals were asked to include full siblings, half-siblings and step-siblings but exclude any that did not live with them during childhood. For each sibling, sex, age, mortality status, and where appropriate age at death were recorded.

### Sample

The household survey was implemented across England (April–July 2013). Questionnaires were completed by individuals in their places of residence under instruction from a professional survey company directed by the research team. All sampled households were sent a letter explaining the study and providing an option to opt out before a surveyor visited. At each household, surveyors asked to speak to the resident who would have the next birthday and was aged 18–69 years. A second opt out opportunity was provided at the door when surveyors again explained the study, emphasizing its voluntary and anonymous nature. Participants could choose to complete the questionnaire through a face-to-face interview using a hand-held computer (with sensitive questions self-completed; *n* = 3852), or self-complete a paper questionnaire (*n* = 158). The questionnaire took an average of 13 min. In order to generate an expected sub-sample of individuals with four or more ACEs of ∼500 (based on a prevalence of ∼12.5% with 4+ ACEs from the pilot study^10^) total sample size was set at 4000. A random probability sample, stratified first by region (*n* = 10, with inner and outer London as two regions) and then small area deprivation, was used to generate a sample representative of the English population. For each region samples were proportionate to their population. Lower super output areas (LSOAs; geographical areas with a population mean of 1500^25^) were categorized into deprivation deciles based on their ranking in the 2010 Index of Multiple Deprivation (IMD; a composite measure including 38 indicators relating to economic, social and housing issues^26^). In each region two LSOAs were randomly selected from each deprivation decile (*n* = 200 LSOAs). From each sampled LSOA, between 40 and 120 addresses were randomly selected from the Postcode Address File^®^ with 16 000 households initially sampled to allow for non-response, ineligibility and non-compliance. For the purposes of analysis individuals were assigned to the quintile of deprivation corresponding to their LSOA of residence.

### Compliance

Following receipt of the study letter, 771 (4.8%) households opted out. Household visits were made all days of the week between 9.30 a.m. and 8.30 p.m. At least three attempted visits were made prior to removing an address, with sampling completed once the target sample size was achieved. Inclusion criteria were resident in a selected LSOA; aged 18–69 years and cognitively able to participate in a face-to-face interview. A total of 9852 households were visited; 7773 were occupied. Of these, 2719 (35.0%) opted out, 1044 (13.4%) were ineligible and 4010 completed a questionnaire. When any respondent opted out or a household contained no eligible individuals the household was replaced by another within the same LSOA to ensure geographical and demographic integrity. The final sample did not differ significantly from the overall English population for deprivation or ethnicity but had a slight over-representation of females and included a higher proportion of individuals aged 60–69 years (and fewer aged 18–29 years).^27^ Cooperation rate^28^ was 59.6% and response rate 43.6% (accounting for vacant houses, refusals and incomplete returns). There was variation in compliance by English region with the cooperation rate varying from 61.6% in East of England to 48.6% in outer London. Ethical approval was obtained from Liverpool John Moores University. The study adhered to the Declaration of Helsinki.

### Analysis

Analyses were undertaken using the PASW Statistics v20 and limited to individuals with complete data relating to ACEs, age, sex, ethnicity and deprivation (*n* = 3885). Across this final sample age distribution was 11.4, 19.5, 20.4, 19.7, 16.6 and 12.4% for age categories 18–24, 25–34, 35–44, 45–54, 55–64 and 65–69 years, respectively. Further, for the purposes of analysis, respondents and their siblings were assigned to a birth cohort either Pre-1969 (age 45+ years) or 1969+ (age 44 years or under). Equally due to small numbers within individual minority ethnic groups, ethnicity was combined into White, Asian and Other. Where individuals did not answer all relevant questions, adjusted sample sizes are presented in tables. Due to highly significant correlations between all ACE types, and consistent with the ACE study methodology elsewhere,^9,10^ a count of how many categories of ACE each individual reported was calculated as a proxy for severity of childhood adversity and classified into four retrospective cohorts (0, 1, 2–3 and 4+ ACES) in order to provide adequate sample sizes for analysis. Siblings (*n* = 6983) were allocated the ACE count, deprivation and ethnicity of corresponding respondents. For cases, life tables used first diagnosis of the relevant condition as the terminating event, and first diagnoses of any included condition for overall disease development. Life tables were calculated for siblings stratified by demographics and ACEs with mortality as the outcome. Differences between strata were examined using Kaplan–Meier analysis. Survival analyses were repeated using Cox regression to identify the independent impact of ACEs on survival. Cox regression used a backwards conditional model with time as a discrete measure. The geographical region of sampling was included in the first strata to account for potential dependencies between observations introduced through sampling in different regions. Gender, ethnicity, birth cohort and deprivation quintile were entered in the second strata. Modelling used birth as the point of origin as all respondent morbidity and sibling mortality could be reported from birth. When combined morbidity and mortality estimated are presented (Fig. 2) the prevalence of morbidity is adjusted to account for individuals removed by deaths. Other analyses used *χ*^2^ and ANOVA tests.

## Results

Across all respondents 46.4% reported suffering at least one ACE and 8.3% ≥4 ACEs (Table 1). All types of child abuse increased with deprivation with, for instance, physical abuse rising from 10.4% of those living in the wealthiest quintile to 18.5% in the poorest. Household stressors were similarly related to deprivation with, for example, living with an alcohol abuser in childhood more than doubling from 5.2 to 12.5% from richest to poorest quintiles (Table 1). Thus, ACE counts showed a strong link with deprivation with proportions suffering ≥4 ACEs nearly tripling from 4.3 to 12.7% across the deprivation gradient. Gender was more weakly associated with ACE risk, with females more likely to report higher levels of ACEs largely due to reporting greater childhood sexual abuse and mental illness in their childhood household. Ethnicity was also associated with ACE count, with Asian ethnicity associated with lowest ACE counts primarily through lower parental separation rates (Table 1). Although there were no significant differences between birth cohort in levels of verbal, physical and sexual abuse experienced as children, greater levels of parental separation, incarceration and household drug and alcohol abuse were reported by those born in 1969 or later. Thus, younger individuals reported higher ACE counts (Table 1). Individuals with more ACEs were significantly more likely to have more siblings (ACEs, mean siblings; 0, 2.11; 1, 2.10; 2–3, 2.26; ≥4, 2.73; *F* = 10.194, *P* < 0.001).

**Table 1:** Socio-demographic distribution of ACEs.

		*Childhood abuse*			*During childhood household included*							*ACE count*			
	**n**	*Verbal*	*Physical*	*Sexual*	*Mental illness*	*Domestic violence*	*Alcohol abuse*	*Incarceration*	*Drug abuse*	*Parental separation*	*Siblings^a^ (mean)*	*0*	*1*	*2–3*	*4+*
All (%)	3885	17.3	14.3	6.2	12.1	12.1	9.1	4.1	3.9	22.6	2.2	53.6	22.7	15.4	8.3
Deprivation quintile															
(least) 1	782	12.7	10.4	5.1	10.6	8.3	5.2	1.4	1.8	16.8	1.9	59.1	24.9	11.6	4.3
2	758	17.2	13.6	5.3	11.5	12.8	9.1	3.3	3.4	21.8	2.0	52.5	25.1	14.8	7.7
3	766	15.5	14.2	5.2	12.9	11.2	8.4	3.0	3.1	22.5	2.1	54.2	23.1	15.9	6.8
4	773	18.4	14.9	7.6	11.3	12.3	10.1	5.8	5.4	24.3	2.3	53.8	18.9	17.3	10.0
(most) 5	806	22.6	18.5	7.4	14.1	15.8	12.5	6.7	5.8	27.7	2.7	48.8	21.5	17.1	12.7
*χ*^2^_trend_		24.758	19.945	6.676	3.527	15.604	23.334	34.784	21.200	26.917	18.905				40.711
*P*		<0.001	<0.001	0.010	0.060	<0.001	<0.001	<0.001	<0.001	<0.001	<0.001				<0.001
Gender															
Male	1749	15.8	14.9	4.5	10.0	11.5	7.9	3.7	3.8	21.4	2.1	54.3	23.8	15.0	6.9
Female	2136	18.5	13.9	7.5	13.8	12.6	10.0	4.4	4.0	23.6	2.3	53.1	21.8	15.6	9.5
*χ*^2^		5.116	0.888	14.729	13.093	1.097	4.994	1.355	0.097	2.802	8.061				9.628
*P*		0.024	0.346	<0.001	<0.001	0.295	0.025	0.244	0.755	0.094	0.005				0.022
Ethnicity^b^															
White	3354	17.7	13.9	6.4	12.6	11.9	9.5	3.9	3.9	23.9	2.0	52.1	23.9	15.7	8.3
Asian	308	10.4	12.7	3.2	7.1	11.7	5.8	2.9	2.9	5.5	3.2	70.1	14.6	10.1	5.2
Other	223	20.2	22.9	7.2	11.2	15.2	8.1	8.1	5.8	26.5	3.4	54.7	14.8	18.4	12.1
*χ*^2^		12.028	14.390	5.136	8.109	2.221	4.738	10.425	2.959	56.670	99.153				49.139
*P*		0.002	0.001	0.077	0.017	0.329	0.094	0.005	0.228	<0.001	<0.001				<0.001
Birth cohort															
1969+	1994	18.4	13.4	5.7	13.0	12.3	10.9	5.9	5.9	29.2	2.0	50.2	23.5	16.4	10.0
Pre-1969	1891	16.1	15.3	6.7	11.1	11.9	7.1	2.1	1.9	15.7	2.3	57.3	21.8	14.3	6.6
*χ*^2^		3.515	2.683	1.668	3.413	0.138	16.910	35.969	40.310	100.766	25.451				26.968
*P*		0.061	0.101	0.197	0.065	0.711	<0.001	<0.001	<0.001	<0.001	<0.001				<0.001

For definition of Adverse Childhood Experiences, see Supplementary data, Box.

^a^Analysis of variance (ANOVA) was used to compare the mean number of siblings who lived with participants during their childhood reported across each demographic group.

^b^Other ethnicity is a combined category of ethnicities each with a prevalence of ≤2.0%.

For respondents, survival analyses were undertaken with earliest diagnosis of each major health condition as the terminating event. All conditions showed lower cumulative survival rates for those with more ACEs, with the steepest ACE gradients for diabetes, respiratory disease and liver/digestive disease. Thus, the cumulative proportion avoiding diagnosis with respiratory disease <age 70 years was 0.907 (±SE, standard error, 0.015) in those with no ACEs but only 0.691 (±SE, 0.104) in those with ≥4 ACEs (Mantel–Cox, *χ*^2^ = 31.731, *P* < 0.001; Supplementary data, Web Table a). To correct for confounding effects of socio-economic and demographic factors, Cox regression was employed (Table 2).

**Table 2:** Changes in risk of disease development with increased history of ACE using Cox regression survival analysis.

		*0 ACEs (ref.)*	*1 ACE*			*2–3 ACEs*			*4+ ACEs*		
	**n**	**P**	*HR*	*95% CIs*	**P**	*HR*	*95% CIs*	**P**	*HR*	*95% CIs*	**P**
Cancer	3881	<0.001	0.75	0.49–1.14	0.171	1.02	0.66–1.59	0.925	2.38	1.48–3.83	<0.001
CVD	3882	0.020	1.24	0.73–2.12	0.424	1.68	0.95–2.94	0.073	3.11	1.56–6.24	0.001
Diabetes type 2	3876	<0.001	1.13	0.80–1.87	0.524	1.22	0.80–1.87	0.346	2.99	1.90–4.72	<0.001
Stroke	3882	0.005	1.63	0.74–3.60	0.229	1.91	0.81–4.48	0.139	5.79	2.43–13.80	<0.001
Respiratory disease	3879	<0.001	1.22	0.77–1.94	0.394	1.83	1.15–2.91	0.010	3.50	2.07–5.91	<0.001
Liver/digestive disease	3879	0.004	1.44	0.99–2.10	0.059	1.45	0.94–2.23	0.093	2.50	1.53–4.08	<0.001
Any disease	3866	<0.001	1.17	0.95–1.42	0.134	1.38	1.11–1.73	0.004	2.76	2.13–3.58	<0.001

ACE, adverse childhood experience (see Supplementary data, Box for definitions); CVD, cardiovascular disease; ref, reference category for Cox regression; HR, adjusted hazard ratio; 95% CI, 95% confidence intervals). See text for additional analytical details.

**Fig. 1:**
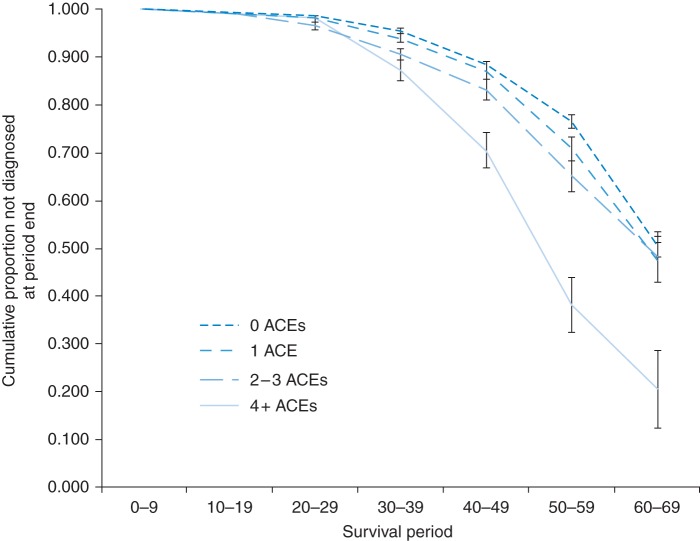
Cumulative proportion of individuals not diagnosed with a major disease with age: unadjusted survival at period end. Respondents reported year of first diagnosis by a doctor or nurse with cancer, CVD, diabetes type 2, stroke, respiratory disease and liver/digestive disease. Details of what constitutes an ACE are given in Supplementary data, Box. See Methods for more details. Kaplan–Meier analysis, variation between ACE categories (Mantel–Cox, *χ*^2^ = 71.671, *P* < 0.001).

For each condition, there was a highly significant difference in rate of disease development between those with no ACEs and those with ≥4 ACEs. For respiratory diseases, differences between no ACES and 2–3 ACEs were also independently significant (Table 2). When all diseases were considered together (Fig. 1), proportions expected to avoid diagnoses of any disease before 70 years dropped from 0.508 (±SE 0.026) in those with no ACEs to only 0.205 (±SE 0.080) in those with ≥4 ACEs. Further, after correcting for demographic confounders, a single ACE did not increase risk of diagnosis of any disease (versus no ACEs), while the impact of 2–3 ACEs and ≥4 ACEs were significant. Thus, compared with individuals with no ACEs, those with 2–3 and 4+ ACEs had a 1.38 and 2.76 times higher rate of being diagnosed with any disease (Table 2) under 70 years of age, respectively.

**Fig. 2:**
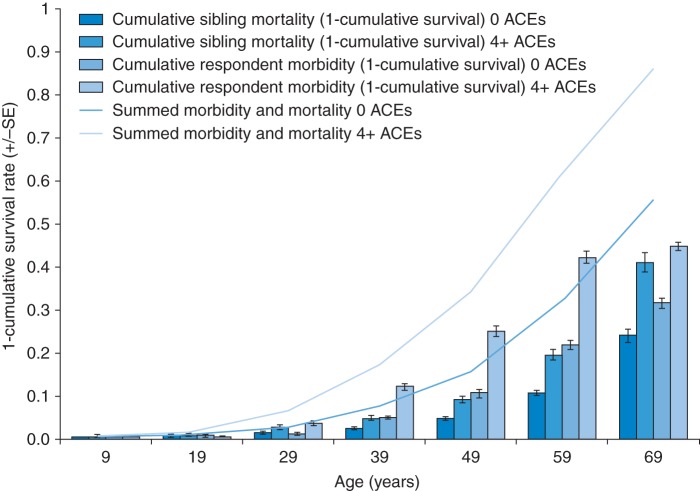
Differences in modelled cumulative morbidity and mortality between deprived males with no or ≥4 adverse childhood experiences. Figures are based on Cox regression models with survival estimates generated at 10-year intervals before age 70 years. Analyses include *n* = 340 (male respondents) and *n* = 849 (siblings) in the most deprived quintile. Morbidity estimates are adjusted to account for proportions of population who have died (see Methods). SE = standard errors, which are shown for morbidity and mortality.

For respondents that lived with one or more sibling in childhood (*n* = 3424, 88.1% of respondents), respondents' ACEs were applied to siblings (Supplementary data, Web Table b) and life tables calculated for sibling mortality survival before 70 years of age. Better survival was strongly associated with lower ACEs, with the cumulative proportion surviving up to 70 years being 0.828 (±SE, 0.015) in those with no ACEs and 0.721 (±SE 0.064) in those with ≥4 ACEs (Mantel–Cox, *χ*^2^ = 19.639, *P* < 0.001; Table 3). Using Cox regression to examine independent impacts on sibling mortality, deprivation, male gender and exposure to ≥4 ACEs remained significantly associated with poor survival (Table 3). The greatest increase in mortality was associated with exposure to ≥4 ACEs (versus no ACEs). Using final Cox regression models, demographically adjusted estimates for morbidity and mortality by ACE exposure were generated for males in the poorest quintile (i.e. the demographic with the highest premature morbidity and mortality; Fig. 2). Here, by age 49 years estimates suggest only 15.79% of males with no ACEs would be affected by chronic morbidity or have died but this rises to 34.21% of those with ≥4 ACEs. By age 69 years these figures have risen to 55.58 and 85.78%, respectively.

**Table 3:** Cumulative survival of siblings stratified by demographics and ACEs.

	*Cumulative survival at period end (standard error)*							*Cox regression*	
	*Age interval (years)*							*HR*	
	*0–9*	*10–19*	*20–29*	*30–39*	*40–49*	*50–59*	*60–69*	*(±95% CI)*	**P**
All	0.997 (0.001)	0.995 (0.001)	0.988 (0.001)	0.980 (0.002)	0.964 (0.003)	0.922 (0.005)	0.813 (0.012)		
ACEs									
0	0.998 (0.001)	0.995 (0.001)	0.990 (0.002)	0.983 (0.002)	0.971 (0.003)	0.927 (0.007)	0.828 (0.015)	Ref.	0.005
1	0.997 (0.001)	0.996 (0.002)	0.989 (0.003)	0.985 (0.003)	0.963 (0.006)	0.922 (0.011)	0.819 (0.025)	1.072 (0.819–1.403)	0.611
2–3	0.999 (0.001)	0.996 (0.002)	0.988 (0.003)	0.978 (0.005)	0.961 (0.007)	0.921 (0.013)	0.782 (0.033)	1.174 (0.872–1.581)	0.291
4+	0.991 (0.004)	0.986 (0.005)	0.970 (0.007)	0.952 (0.010)	0.929 (0.014)	0.887 (0.022)	0.721 (0.064)	1.965 (1.386–2.786)	<0.001
Gender									
Male	0.996 (0.001)	0.994 (0.001)	0.984 (0.002)	0.972 (0.003)	0.954 (0.004)	0.898 (0.008)	0.785 (0.017)	Ref.	
Female	0.998 (0.001)	0.996 (0.001)	0.993 (0.002)	0.989 (0.002)	0.976 (0.003)	0.947 (0.006)	0.844 (0.016)	0.566 (0.455–0.704)	<0.001
Deprivation quintile									
(least)1	0.999 (0.001)	0.998 (0.001)	0.992 (0.003)	0.986 (0.004)	0.977 (0.005)	0.944 (0.010)	0.880 (0.022)	Ref.	0.013
2	0.999 (0.001)	0.998 (0.001)	0.994 (0.002)	0.988 (0.003)	0.963 (0.007)	0.913 (0.012)	0.797 (0.026)	1.599 (1.106–2.312)	0.013
3	0.994 (0.002)	0.988 (0.003)	0.983 (0.004)	0.979 (0.004)	0.969 (0.005)	0.939 (0.010)	0.810 (0.027)	1.426 (0.979–2.078)	0.065
4	0.998 (0.001)	0.995 (0.002)	0.987 (0.003)	0.978 (0.004)	0.969 (0.006)	0.916 (0.013)	0.791 (0.029)	1.541 (1.056–2.248)	0.025
(most) 5	0.996 (0.001)	0.995 (0.002)	0.986 (0.003)	0.972 (0.005)	0.947 (0.007)	0.899 (0.012)	0.796 (0.026)	1.858 (1.296–2.663)	<0.001
Ethnicity									
White	0.997 (0.001)	0.995 (0.001)	0.988 (0.001)	0.980 (0.002)	0.965 (0.003)	0.921 (0.005)	0.814 (0.012)	Ref.	0.644
Asian	0.999 (0.001)	0.996 (0.002)	0.988 (0.004)	0.986 (0.005)	0.968 (0.009)	0.936 (0.018)	0.829 (0.053)	0.927 (0.596–1.441)	0.736
Other	0.998 (0.002)	0.998 (0.002)	0.987 (0.006)	0.972 (0.009)	0.943 (0.016)	0.919 (0.023)	0.780 (0.067)	1.218 (0.770–1.927)	0.399

HR, adjusted hazard ratio; Ref., reference category; ACE, adverse childhood experience; ±95% CI, 95% confidence interval. Sample size for all analyses, *n* = 6983. See text for additional analytical details.

## Discussion

### Main findings of this study

ACE counts were positively associated with deprivation (Table 1) and, like other studies,^29–31^ deprivation was associated with premature mortality (Table 3). However, even within similar ecologies individuals experience radically different childhoods. Here, in England's most deprived quintile, 48.8% of individuals reported no ACEs with 12.7% reporting ≥4. Results provide evidence of the importance of these early life experiences in setting individuals' lifetime health trajectories. Across the life course risks of each individual disease <70 years of age were significantly higher in those with ≥4 ACEs compared with those with none, even after accounting for the impacts of deprivation (Table 2). Thus, individuals with ≥4 ACEs (versus no ACEs) have increased rates of developing NCDs ranging from 2.38 times for cancer to 5.79 for stroke. For all diseases combined, risks increased in those with ≥2 ACEs (Table 2). These impacts should be considered on top of the impacts of ACEs on premature mortality, with rates of premature mortality being 1.97 times higher in those with ≥4 ACEs compared with those with none (Table 3). Such higher rates will have disproportionately removed individuals from high ACE categories; in part masking their impact on morbidity.

### What is already known on this topic

ACE studies have been used globally to examine the relationships between early life stressors, the development of health-harming behaviours and, to a lesser extent, risks of NCDs in later life. Exposure to childhood trauma is now known to cause immediate physical damage (e.g. injury) but also introduce lasting biological and psychological changes. Thus, childhood trauma can alter brain development as well as immunological and endocrine functions (e.g. heightened nervous and immunological activity, dulled emotional response, increased propensity for aggression).^8,16,32–34^ While consistent with adaption to harsh environments, such allostatic loads (i.e. physiological responses to environmental change) add to wear and tear on physiological systems and consequently can expedite development of poor health with age.^16^ These factors combine with other effects of childhood stressors. Thus, both prospective and retrospective studies have identified links between ACEs and poorer control over calorie intake leading to obesity.^35^ Further, multiple studies show exposure to ACEs increases propensity for behaviours linked to development of NCDs including alcohol, tobacco and drug use. Such behaviours, obesity and other impacts of allostatic overload all contribute to increased risks of premature morbidity in ACE sufferers.^16,27^

### What this study adds

Retrospective ACE studies can be a rapid and effective mechanism for identifying the costs of childhood adversity to health and health-care systems and to advocate for investment in effective early years prevention. Here, we have quantified the relationship between ACEs and morbidity and mortality across the life course and provided a framework for the potential reduction in ill health and early death that might be achieved if ACEs are prevented. By incorporating novel questions on sibling mortality in a standard retrospective ACE survey we identified nearly double the rate of premature mortality up to the age of 70 years in those with ≥4 ACEs (versus none). Findings highlight a strong life-course relationship between ACEs and both premature mortality and morbidity in England which is independent of deprivation and a major, but avoidable, cause of health harms and health-care costs. Critically, these occur in addition to the physical and mental harms individuals suffer as children and the broader costs to individuals and society through poorer social outcomes and economic performance.

### Limitations of this study

Although our sample aimed to be representative of the English population, a residence-based sampling framework excludes those who are homeless, resident in health-care settings or incarcerated; all of whom may have substantive ACE counts.^36–38^ Further, ACE studies can be limited by non-participation, recall accuracy and individual propensity to exaggerate or omit ACEs. Although participation bias cannot be ruled out, participation rates were comparable to other national surveys^39,40^ and ACE questions were completed by the vast majority of participants. However, cooperation rates varied by English Region and it was not possible to examine whether geographic or demographic variations in compliance impacted results. Respondents self-reported health conditions and while only conditions diagnosed by a doctor or nurse were recorded, it is not possible to measure the extent of undiagnosed morbidity. While siblings were limited to those living with respondents during childhood, differences from respondents in age, gender and other factors may have resulted in siblings and respondents experiencing different ACEs. However, many ACEs are likely to be common across siblings and any child being abused in a family is a risk factor for abuse of the others.^21,22^ Current deprivation was also not available for siblings and instead was based on respondents. However, social mobility is relatively limited in England, restricting lifetime migration between deprivation and affluence.^41^ Some sibling mortality may not be known to respondents and for both respondents with no siblings or families where all individuals have died this method will not capture a measure of mortality. Consequently, mortality related to ACEs will inevitably be underreported using siblings as a proxy measure. While we employed analytical methodologies that accommodate data censoring in survival analyses, younger individuals are increasingly censored from survival estimates with increasing age. Although this could result in HRs between ACE categories changing with increasing time, parallel log–log plots for survival suggested that proportional hazards assumptions were valid for both disease and mortality analyses.^42^

## Conclusions

By extending ACE survey methodologies we have added a measure of premature mortality to associations between childhood stressors and the development of morbidity across the life course. Four NCDs (cardiovascular disease, cancer, chronic respiratory disease and diabetes) now account for over half of global deaths.^43^ Despite increasing evidence that poor early life environments are a major contributor to the development of such disease, health investments in better quality childhoods are negligible compared with those in treatment services.^44^ Changing this balance requires better understanding of the impact of ACEs, a realignment of health policy and coordinated multi-sectoral and targeted working. Thus, advocacy for change necessitates greater public and professional understanding of the health consequences of ACEs, as well as their impact on employment, aggression and even the parenting skills of those who suffered ACEs. Health policy at local, national and international levels should set out sustainable investment at scale in those evidence-based interventions known to reduce ACEs through supporting nurturing childhoods.^45^ Implementing such interventions is not limited to health service activity but requires shared objectives and working with criminal justice, social and education systems as well as integrated research and intelligence systems to target interventions and monitor progress. While many ACEs are disproportionately found in poorer communities, results here identify that even affluent communities are not free from ACEs. However, the association between larger family sizes (more siblings) and higher ACE counts suggests that populations most at risk of adverse childhoods may be growing disproportionately. Consequently, while universal interventions are required these should be deployed proportionate to need. Although such interventions are affordable, increasingly the long-term behavioural, social and chronic health costs that arise from leaving ACEs unaddressed are not.

## Supplementary data

Supplementary data are available at the *Journal of Public Health* online.

## Funding

The study was co-funded through Higher Education Funding Council for England and Public Health Observatory (National Health Services) support provided to Liverpool John Moores University.

## Supplementary Material

Supplementary Data
