# Supplementary material for: Measuring mortality and the burden of adult disease associated with adverse childhood experiences in England: a national survey
Source: J Public Health (Oxf). 2014 Aug 30;37(3):445–54. doi: 10.1093/pubmed/fdu065 (PMC4552010; doi:10.1093/pubmed/fdu065)
Supplement: Supplementary Data [file supp_37_3_445__index.html]

Measuring mortality and the burden of adult disease associated with adverse childhood experiences in England: a national survey — Measuring mortality and the burden of adult disease associated with adverse childhood experiences in England: a national survey — Supplementary Data 

# Measuring mortality and the burden of adult disease associated with adverse childhood experiences in England: a national survey

## Supplementary Data

Supplementary Data

**Files in this Data Supplement:**

- Supplementary Data - Docx file
