# Supplementary material for: Measuring mortality and the burden of adult disease associated with adverse childhood experiences in England: a national survey
Source: J Public Health (Oxf). 2014 Aug 30;37(3):445–54. doi: 10.1093/pubmed/fdu065 (PMC4552010; doi:10.1093/pubmed/fdu065)
Supplement: Supplementary Data [file supp_fdu065_fdu065supp.docx]

**Web Box and Tables**

| **Web Box: Adverse Childhood Experiences (ACEs)** | | | |
| --- | --- | --- | --- |
| All ACE questions were preceded by the statement “While you were growing up, before the age of 18…” | | | |
| **ACE** | **Question** | | **Response** |
| Parental separation | | Were your parents ever separated or divorced? | Yes |
| Domestic violence | | How often did your parents or adults in your home ever slap, hit, kick, punch or beat each other up? | Once or more than once |
| Physical abuse | | How often did a parent or adult in your home ever hit, beat, kick or physically hurt you in any way? This does not include gentle smacking for punishment | Once or more than once |
| Verbal abuse | | How often did a parent or adult in your home ever swear at you, insult you, or put you down? | More than once |
| Sexual abuse | | How often did anyone at least 5 years older than you (including adults) ever touch you sexually? | Once or more than once to any of the three questions |
|  |  | How often did anyone at least 5 years older than you (including adults) try to make you touch them sexually? |  |
|  |  | How often did anyone at least 5 years older than you (including adults) force you to have any type of sexual intercourse (oral, anal or vaginal)? |  |
| Mental illness | | Did you live with anyone who was depressed, mentally ill or suicidal? | Yes |
| Alcohol abuse | | Did you live with anyone who was a problem drinker or alcoholic? | Yes |
| Drug abuse | | Did you live with anyone who used illegal street drugs or who abused prescription medications? | Yes |
| Incarceration | | Did you live with anyone who served time or was sentenced to serve time in a prison or young offenders institution? | Yes |

**Web Table a. Cumulative proportion of individuals who have not been diagnosed with each disease at the end of each age period by ACE history**

|  |  |  | Cumulative proportion not diagnosed at period end (years) | | | | | | | Kaplan-Meier^+^ | |
| --- | --- | --- | --- | --- | --- | --- | --- | --- | --- | --- | --- |
| Age | |  | 0-9 | 10-19 | 20-29 | 30-39 | 40-49 | 50-59 | 60-69 | *X*^2^ | P |
| **Cancer** | **All** |  | **1.000** | **1.000** | **0.996** | **0.987** | **0.963** | **0.924** | **0.829** |  |  |
|  |  | SE+/- | 0.000 | 0.000 | 0.001 | 0.002 | 0.004 | 0.007 | 0.015 |  |  |
|  | **ACEs** | **0** | **1.000** | **1.000** | **0.996** | **0.990** | **0.965** | **0.933** | **0.812** | 19.209 | <0.001 |
|  |  | SE+/- | 0.000 | 0.000 | 0.001 | 0.002 | 0.005 | 0.008 | 0.022 |  |  |
|  |  | **1** | **1.000** | **1.000** | **0.996** | **0.991** | **0.977** | **0.942** | **0.871** |  |  |
|  |  | SE+/- | 0.000 | 0.000 | 0.002 | 0.004 | 0.007 | 0.013 | 0.028 |  |  |
|  |  | **2-3** | **1.000** | **1.000** | **0.996** | **0.980** | **0.955** | **0.908** | **0.875** |  |  |
|  |  | SE+/- | 0.000 | 0.000 | 0.003 | 0.007 | 0.012 | 0.020 | 0.030 |  |  |
|  |  | **4+** | **1.000** | **1.000** | **0.996** | **0.963** | **0.923** | **0.799** | **0.729** |  |  |
|  |  | SE+/- | 0.000 | 0.000 | 0.004 | 0.013 | 0.021 | 0.047 | 0.079 |  |  |
| **CVD** | **All** |  | **1.000** | **1.000** | **1.000** | **0.998** | **0.986** | **0.955** | **0.897** |  |  |
|  |  |  | 0.000 | 0.000 | 0.000 | 0.001 | 0.003 | 0.006 | 0.013 |  |  |
|  | **ACEs** | **0** | **1.000** | **1.000** | **1.000** | **0.999** | **0.987** | **0.968** | **0.911** | 11.932 | 0.008 |
|  |  | SE+/- | 0.000 | 0.000 | 0.000 | 0.001 | 0.003 | 0.006 | 0.016 |  |  |
|  |  | **1** | **1.000** | **1.000** | **1.000** | **0.997** | **0.987** | **0.952** | **0.900** |  |  |
|  |  | SE+/- | 0.000 | 0.000 | 0.000 | 0.002 | 0.005 | 0.013 | 0.025 |  |  |
|  |  | **2-3** | **1.000** | **1.000** | **1.000** | **0.997** | **0.990** | **0.931** | **0.865** |  |  |
|  |  | SE+/- | 0.000 | 0.000 | 0.000 | 0.003 | 0.006 | 0.019 | 0.036 |  |  |
|  |  | **4+** | **1.000** | **1.000** | **1.000** | **0.995** | **0.963** | **0.889** | **0.808** |  |  |
|  |  | SE+/- | 0.000 | 0.000 | 0.000 | 0.005 | 0.016 | 0.039 | 0.085 |  |  |
| **Diabetes** | **All** |  | **1.000** | **1.000** | **0.999** | **0.990** | **0.967** | **0.911** | **0.807** |  |  |
| **Type 2** |  |  | 0.000 | 0.000 | 0.001 | 0.002 | 0.004 | 0.008 | 0.016 |  |  |
|  | **ACEs** | **0** | **1.000** | **1.000** | **0.999** | **0.990** | **0.970** | **0.928** | **0.835** | 33.040 | <0.001 |
|  |  | SE+/- | 0.000 | 0.000 | 0.001 | 0.003 | 0.005 | 0.009 | 0.020 |  |  |
|  |  | **1** | **1.000** | **1.000** | **1.000** | **0.998** | **0.972** | **0.928** | **0.779** |  |  |
|  |  | SE+/- | 0.000 | 0.000 | 0.000 | 0.002 | 0.008 | 0.015 | 0.037 |  |  |
|  |  | **2-3** | **1.000** | **1.000** | **0.996** | **0.988** | **0.966** | **0.883** | **0.817** |  |  |
|  |  | SE+/- | 0.000 | 0.000 | 0.003 | 0.005 | 0.010 | 0.023 | 0.038 |  |  |
|  |  | **4+** | **1.000** | **1.000** | **1.000** | **0.971** | **0.932** | **0.723** | **0.578** |  |  |
|  |  | SE+/- | 0.000 | 0.000 | 0.000 | 0.011 | 0.021 | 0.055 | 0.102 |  |  |
| **Stroke** | **All** |  | **1.000** | **1.000** | **0.999** | **0.998** | **0.992** | **0.980** | **0.953** |  |  |
|  |  |  | 0.000 | 0.000 | 0.001 | 0.001 | 0.002 | 0.004 | 0.009 |  |  |
|  | **ACEs** | **0** | **1.000** | **1.000** | **1.000** | **0.999** | **0.996** | **0.990** | **0.961** | 19.596 | <0.001 |
|  |  | SE+/- | 0.000 | 0.000 | 0.000 | 0.001 | 0.002 | 0.004 | 0.011 |  |  |
|  |  | **1** | **1.000** | **1.000** | **0.999** | **0.997** | **0.992** | **0.976** | **0.955** |  |  |
|  |  | SE+/- | 0.000 | 0.000 | 0.001 | 0.002 | 0.004 | 0.009 | 0.017 |  |  |
|  |  | **2-3** | **1.000** | **1.000** | **0.996** | **0.993** | **0.986** | **0.980** | **0.946** |  |  |
|  |  | SE+/- | 0.000 | 0.000 | 0.003 | 0.004 | 0.006 | 0.009 | 0.025 |  |  |
|  |  | **4+** | **1.000** | **1.000** | **1.000** | **1.000** | **0.976** | **0.885** | **0.885** |  |  |
|  |  | SE+/- | 0.000 | 0.000 | 0.000 | 0.000 | 0.014 | 0.041 | 0.041 |  |  |
| **Respiratory** | **All** |  | **1.000** | **0.998** | **0.996** | **0.985** | **0.974** | **0.939** | **0.884** |  |  |
| **Disease** |  |  | 0.000 | 0.001 | 0.001 | 0.002 | 0.003 | 0.006 | 0.013 |  |  |
|  | **ACEs** | **0** | **1.000** | **0.999** | **0.998** | **0.991** | **0.980** | **0.956** | **0.907** | 31.731 | <0.001 |
|  |  | SE+/- | 0.000 | 0.001 | 0.001 | 0.002 | 0.004 | 0.007 | 0.015 |  |  |
|  |  | **1** | **1.000** | **0.999** | **0.996** | **0.984** | **0.980** | **0.933** | **0.903** |  |  |
|  |  | SE+/- | 0.000 | 0.001 | 0.002 | 0.005 | 0.006 | 0.014 | 0.022 |  |  |
|  |  | **2-3** | **1.000** | **0.998** | **0.992** | **0.977** | **0.966** | **0.918** | **0.821** |  |  |
|  |  | SE+/- | 0.000 | 0.002 | 0.004 | 0.007 | 0.010 | 0.019 | 0.041 |  |  |
|  |  | **4+** | **1.000** | **0.994** | **0.990** | **0.967** | **0.919** | **0.844** | **0.691** |  |  |
|  |  | SE+/- | 0.000 | 0.004 | 0.006 | 0.012 | 0.022 | 0.041 | 0.104 |  |  |
| **Liver/** | **All** |  | **1.000** | **0.999** | **0.992** | **0.977** | **0.958** | **0.925** | **0.871** |  |  |
| **Digestive** |  |  | 0.000 | 0.001 | 0.002 | 0.003 | 0.004 | 0.007 | 0.013 |  |  |
| **Disease** | **ACEs** | **0** | **1.000** | **1.000** | **0.994** | **0.985** | **0.970** | **0.943** | **0.883** | 17.782 | <0.001 |
|  |  | SE+/- | 0.000 | 0.000 | 0.002 | 0.003 | 0.005 | 0.008 | 0.017 |  |  |
|  |  | **1** | **1.000** | **0.998** | **0.991** | **0.969** | **0.948** | **0.913** | **0.872** |  |  |
|  |  | SE+/- | 0.000 | 0.002 | 0.003 | 0.007 | 0.010 | 0.015 | 0.024 |  |  |
|  |  | **2-3** | **1.000** | **0.997** | **0.984** | **0.969** | **0.951** | **0.905** | **0.873** |  |  |
|  |  | SE+/- | 0.000 | 0.002 | 0.005 | 0.008 | 0.011 | 0.019 | 0.029 |  |  |
|  |  | **4+** | **1.000** | **1.000** | **0.993** | **0.965** | **0.901** | **0.845** | **0.691** |  |  |
|  |  | SE+/- | 0.000 | 0.000 | 0.005 | 0.012 | 0.025 | 0.039 | 0.103 |  |  |

ACE = Adverse Childhood Experiences (see Web Box for definitions). CVD = Cardiovascular disease. SE = Standard Error. ^+^X^2^ and P value from Mantel-Cox comparison between ACE categories.

**Web Table b. Socio-demographic distribution of Adverse Childhood Experiences in siblings^+^ of respondents reporting to English national survey**

|  |  |  | **Childhood abuse** | | |  | **During childhood household included** | | | | | |  | **ACE Count** | | | | | | |
| --- | --- | --- | --- | --- | --- | --- | --- | --- | --- | --- | --- | --- | --- | --- | --- | --- | --- | --- | --- | --- |
|  |  | n | Verbal | Physical | Sexual |  | Mental illness | Domestic violence | Alcohol abuse | Incarc-eration | Drug abuse | Parental separation |  | 0 | 1 | | 2-3 | | 4+ | |
| **Controls - Prevalence (%)** | | 6983 | 17.2 | 15.6 | 6.1 |  | 10.8 | 13.9 | 9.3 | 4.4 | 3.9 | 20.5 |  | 54.1 | | 22.2 | | 15.4 | | 8.3 |
| **Deprivation** | *(least) 1* | 1267 | 11.7 | 11.8 | 5.1 |  | 9.4 | 9.8 | 5.6 | 1.2 | 2.0 | 14.8 |  | 58.8 | | 25.7 | | 11.8 | | 3.8 |
| **quintile** | *2* | 1281 | 15.8 | 13.7 | 5.0 |  | 9.0 | 14.8 | 8.2 | 2.7 | 3.1 | 17.6 |  | 55.1 | | 25.8 | | 12.3 | | 6.8 |
|  | *3* | 1360 | 17.3 | 16.8 | 5.4 |  | 11.4 | 13.2 | 9.1 | 3.4 | 3.2 | 19.8 |  | 54.1 | | 22.0 | | 16.6 | | 7.3 |
|  | *4* | 1427 | 17.7 | 15.7 | 8.1 |  | 10.8 | 14.5 | 11.0 | 7.4 | 5.5 | 23.1 |  | 52.5 | | 18.8 | | 18.0 | | 10.7 |
|  | *(most) 5* | 1648 | 22.1 | 18.9 | 6.7 |  | 12.8 | 16.3 | 11.8 | 6.6 | 5.0 | 25.7 |  | 51.0 | | 19.7 | | 17.5 | | 11.8 |
|  | *X^2trend^* |  | 53.123 | 28.051 | 9.401 |  | 11.375 | 18.856 | 38.393 | 79.278 | 26.979 | 64.167 |  | 81.143 | | | | | | |
|  | P |  | <0.001 | <0.001 | 0.002 |  | 0.001 | <0.001 | <0.001 | <0.001 | <0.001 | <0.001 |  | <0.001 | | | | | | |
| **Sex** | *Male* | 3624 | 16.4 | 15.9 | 6.1 |  | 10.4 | 13.8 | 9.5 | 5.0 | 4.2 | 20.2 |  | 54.4 | | 22.0 | | 15.5 | | 8.2 |
|  | *Female* | 3359 | 18.1 | 15.3 | 6.1 |  | 11.2 | 13.9 | 9.2 | 3.8 | 3.5 | 21.0 |  | 53.8 | | 22.4 | | 15.4 | | 8.5 |
|  | *X^2^* |  | 3.228 | 0.422 | 0.012 |  | 1.055 | 0.027 | 0.215 | 6.615 | 1.984 | 0.662 |  | 0.445 | | | | | | |
|  | P |  | 0.072 | 0.516 | 0.914 |  | 0.304 | 0.870 | 0.643 | 0.010 | 0.159 | 0.416 |  | 0.931 | | | | | | |
| **Ethnicity** | *White* | 5722 | 17.7 | 15.3 | 6.3 |  | 11.3 | 13.8 | 10.0 | 4.2 | 3.9 | 22.2 |  | 52.1 | | 23.6 | | 15.9 | | 8.5 |
|  | *Asian* | 797 | 12.3 | 14.1 | 2.9 |  | 6.4 | 13.6 | 5.9 | 3.4 | 2.8 | 4.5 |  | 69.0 | | 14.6 | | 11.3 | | 5.1 |
|  | *Other*† | 464 | 19.8 | 22.0 | 8.6 |  | 11.6 | 15.1 | 7.3 | 8.6 | 5.8 | 27.4 |  | 52.8 | | 18.1 | | 17.0 | | 12.1 |
|  | *X^2^* |  | 16.702 | 16.223 | 20.115 |  | 18.112 | 0.662 | 16.140 | 21.701 | 7.355 | 148.596 |  | 94.396 | | | | | | |
|  | P |  | <0.001 | <0.001 | <0.001 |  | <0.001 | 0.718 | <0.001 | <0.001 | 0.025 | <0.001 |  | <0.001 | | | | | | |
| **Birth** | *1969+* | 3319 | 18.1 | 14.0 | 5.0 |  | 11.7 | 13.0 | 10.5 | 6.4 | 6.2 | 26.0 |  | 52.0 | | 22.6 | | 15.8 | | 9.6 |
| **cohort** | *Pre 1969* | 3664 | 16.4 | 17.0 | 7.1 |  | 10.0 | 14.7 | 8.3 | 2.6 | 1.8 | 15.6 |  | 55.9 | | 21.8 | | 15.1 | | 7.2 |
|  | *X^2^* |  | 3.677 | 12.069 | 14.079 |  | 5.592 | 4.069 | 9.850 | 58.352 | 89.369 | 113.860 |  | 17.759 | | | | | | |
|  | P |  | 0.055 | 0.001 | <0.001 |  | 0.018 | 0.004 | 0.002 | <0.001 | <0.001 | <0.001 |  | <0.001 | | | | | | |

ACE = Adverse Childhood Experience. ^+^Siblings included in the analyses are those reported by respondents to the national survey as co-habiting with them for all or part of their childhood. ACEs and ethnicity for siblings are derived directly from those reported by corresponding respondents (see methods). Other demographics (age, gender) were reported for each sibling by respondents. †Other Ethnicity is a combined category of ethnicities each with a prevalence of ≤2.0%. See methods for component ethnicities.
